# Supplementary material for: The interactome of intact mitochondria by cross-linking mass spectrometry provides evidence for coexisting respiratory supercomplexes
Source: Mol Cell Proteomics. 2017 Dec 8;17(2):216–32. doi: 10.1074/mcp.RA117.000470 (PMC5795388; doi:10.1074/mcp.RA117.000470)
Supplement: Supplemental Data [file supp_RA117.000470_133922_0_supp_23978_fzffwf.zip › spectra_annotation/mito_spectra_annotation/106-1-5-1-6-1.pdf]

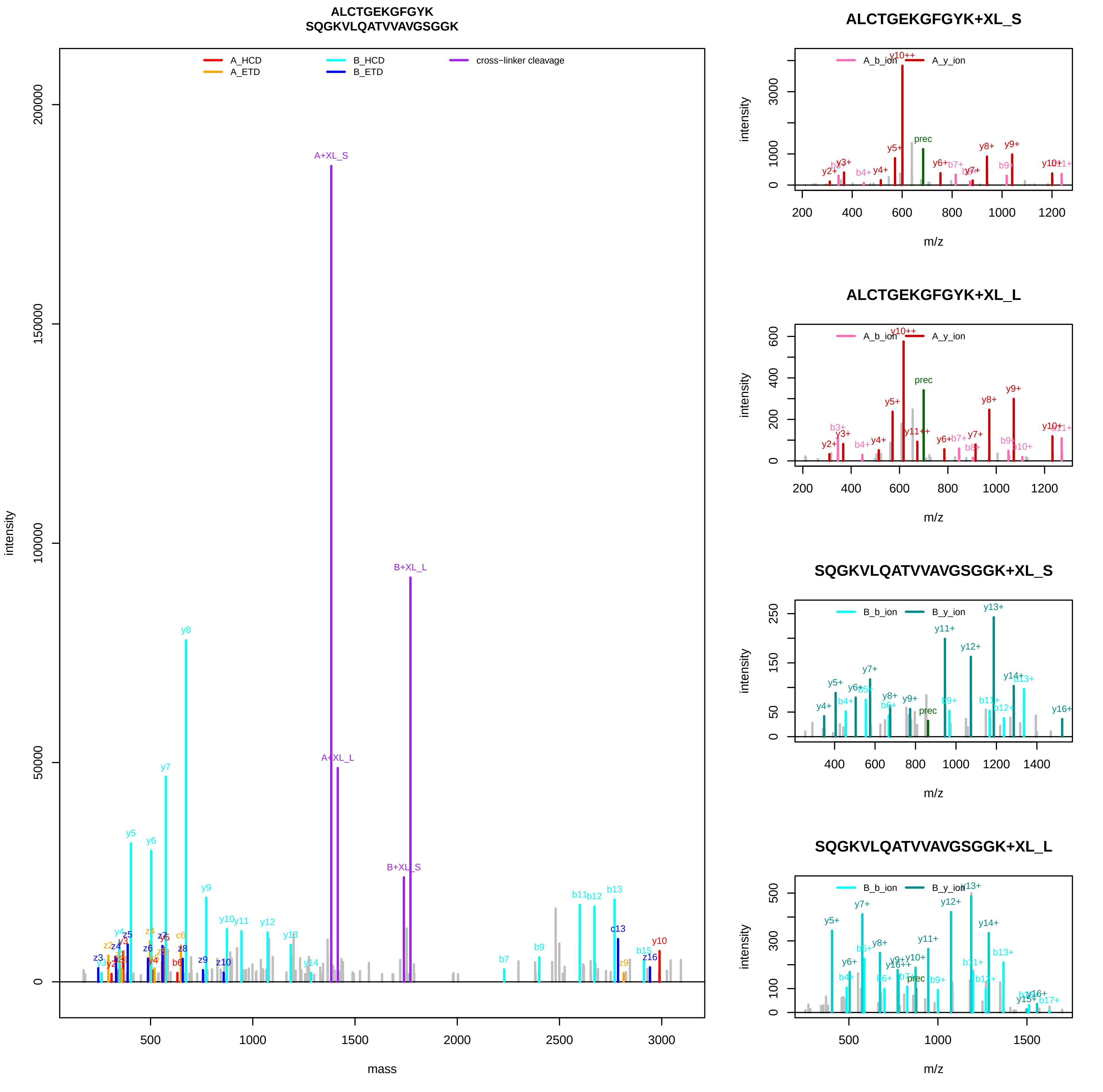

ALCTGEKGFGYK
SQGKVLQATVVAVGSGGK
A_HCD
A_ETD
B_HCD
B_ETD
cross−linker cleavage
A+XL_S
B+XL_L
A+XL_L
B+XL_S
y8
y7
y5
y6
y9
y10
y11
y12
y13
y14
b7
b9
b11
b12
b13
b15
c13
z16
z9
y10
c6
z8
z9
z10
intensity
mass
0
50000
100000
150000
200000
500
1000
1500
2000
2500
3000
ALCTGEKGFGYK+XL_S
A_b_ion
A_y_ion
y10++
prec
y2+
y3+
y4+
y5+
y6+
y7+
y8+
y9+
y10+
b4+
b7+
b9+
intensity
m/z
ALCTGEKGFGYK+XL_L
y11++
b3+
b8+
b10+
SQGKVLQATVVAVGSGGK+XL_S
B_b_ion
B_y_ion
y4+
y5+
y6+
y7+
y8+
y9+
y11+
y12+
y13+
y14+
y16+
b4+
b5+
b6+
b9+
b11+
b12+
b13+
SQGKVLQATVVAVGSGGK+XL_L
y15+
y16+
b17+
y16++
y10+
